# Biochemical, Structural and Molecular Dynamics Analyses of the Potential Virulence Factor RipA from *Yersinia pestis*

**DOI:** 10.1371/journal.pone.0025084

**Published:** 2011-09-26

**Authors:** Rodrigo Torres, Robert V. Swift, Nicholas Chim, Nicole Wheatley, Benson Lan, Brian R. Atwood, Céline Pujol, Banu Sankaran, James B. Bliska, Rommie E. Amaro, Celia W. Goulding

**Affiliations:** 1 Department of Molecular Biology and Biochemistry, University of California Irvine, Irvine, California, United States of America; 2 Department of Pharmaceutical Sciences, University of California Irvine, Irvine, California, United States of America; 3 Departments of Computer Science and Chemistry, University of California Irvine, Irvine, California, United States of America; 4 Department of Molecular Genetics and Microbiology and Center for Infectious Diseases, State University of New York, Stony Brook, New York, United States of America; 5 Lawrence Berkeley National Laboratory, Berkeley Center for Structural Biology, Berkeley, California, United States of America; University of Glasgow, United Kingdom

## Abstract

Human diseases are attributed in part to the ability of pathogens to evade the eukaryotic immune systems. A subset of these pathogens has developed mechanisms to survive in human macrophages. *Yersinia pestis*, the causative agent of the bubonic plague, is a predominately extracellular pathogen with the ability to survive and replicate intracellularly. A previous study has shown that a novel *rip* (required for intracellular proliferation) operon (*ripA*, *ripB* and *ripC*) is essential for replication and survival of *Y. pestis* in postactivated macrophages, by playing a role in lowering macrophage-produced nitric oxide (NO) levels. A bioinformatics analysis indicates that the *rip* operon is conserved among a distally related subset of macrophage-residing pathogens, including *Burkholderia* and *Salmonella* species, and suggests that this previously uncharacterized pathway is also required for intracellular survival of these pathogens. The focus of this study is *ripA*, which encodes for a protein highly homologous to 4-hydroxybutyrate-CoA transferase; however, biochemical analysis suggests that RipA functions as a butyryl-CoA transferase. The 1.9 Å X-ray crystal structure reveals that RipA belongs to the class of Family I CoA transferases and exhibits a unique tetrameric state. Molecular dynamics simulations are consistent with RipA tetramer formation and suggest a possible gating mechanism for CoA binding mediated by Val227. Together, our structural characterization and molecular dynamic simulations offer insights into acyl-CoA specificity within the active site binding pocket, and support biochemical results that RipA is a butyryl-CoA transferase. We hypothesize that the end product of the *rip* operon is butyrate, a known anti-inflammatory, which has been shown to lower NO levels in macrophages. Thus, the results of this molecular study of *Y. pestis* RipA provide a structural platform for rational inhibitor design, which may lead to a greater understanding of the role of RipA in this unique virulence pathway.

## Introduction

*Yersinia pestis* is the causative agent of the bubonic plague, a fatal disease that has resulted in three major pandemics throughout history, and was responsible for killing approximately half the population of Europe in the 14^th^ century. Although outbreaks of the plague have decreased greatly within the last two centuries, the disease is still endemic in regions of North and South America, Africa and Asia, particularly in rural areas where *Y. pestis* infection can spread and be lethal if not properly treated with antibiotics. In more recent times, *Y. pestis* has emerged as a biological weapon, especially in light of its propensity to evolve multidrug resistance [1]. Additionally, within the genus *Yersinia*, two enteropathogens, *Y. pseudotuberculosis* and *Y. enterocolitica* cause a broad range of gastrointestinal diseases [2] resulting in at least 30,000 documented cases per year. Thus, understanding the mechanisms of pathogenesis of *Yersinia* remains an important goal.

*Y. pestis* extracellular pathogenicity has been well established [3]. However, *Y. pestis* is a facultative intracellular pathogen that is capable of surviving and replicating in macrophages, as such it has been suggested that *Y. pestis* is initially phagocytosed by macrophages before it escapes and replicates in the extracellular environment. Studies have demonstrated that the *pgm* locus, previously associated with a critical iron transport system, is important for *Y. pestis* replication in interferon-γ (INF-γ) treated (postactivated) macrophages [4]. Interestingly, *Y. pestis* replication in postactivated macrophages is coupled with reduced toxic nitric oxide (NO) levels although INF-g up-regulates macrophage inducible NO synthase (iNOS) expression. Furthermore, a *Y. pestis *Δ*pgm* mutant does not survive in postactivated wild-type macrophages, while it can replicate in postactivated iNOS^−/−^ macrophages, suggesting that killing of the Δ*pgm* mutant is NO-dependent. Two previously unannotated genes, *ripB* and *ripA*, within the *pgm* locus were further identified to be essential for intracellular *Y. pestis* replication. Deletion of either *ripB* or *ripA* alone resulted in mutants that showed an inability to replicate in postactivated macrophages, which correlated with their inability to decrease NO levels within the macrophage, suggesting that *ripB* and *ripA* are directly or indirectly responsible for lowering macrophage-produced NO levels. Thus, *ripA* and *ripB* along with a third gene *ripC*, constitute a novel virulence operon designated *rip* (required for intracellular proliferation), thought to be important in intracellular replication of *Y. pestis* in macrophages. In order to better understand the mechanism of action of the *rip* operon, investigation into the proteins encoded by the *rip* genes is essential.

The *ripA* gene encodes for a protein highly homologous to 4-hydroxybutyrate-CoA transferase (4-HB-CoAT), which belongs to a superfamily of CoA transferases responsible for the transfer of the CoAS^−^ anion from a donor CoA thioester to an acceptor free acid. 4-HB-CoAT is further proposed to belong to the Family I CoA transferases [5], one of the three distinct sub-families defined by their structure and mechanism [6]. Structurally, Family I CoA transferases contain two distinct subunits of approximately equal amino acid length with similar α/β-folds, which assemble as α_2_β_2_ or α_4_β_4_ oligomers [7], [8]; 4-HB-CoAT [5], succinyl-CoA:3-oxoacid transferase (SCOT) [9] and *Escherichia coli* YdiF [10] form a subset of this group, whereby the a and b subunits are connected by a linker region to form one polypeptide chain. Mechanistically, Family I CoA transferases share a common ping-pong mechanism whereby the first half of the reaction involves a conserved catalytic glutamate in the active site that forms a covalent CoA-thioester intermediate [11]. Specifically, the glutamate side chain attacks the CoA-donor carbonyl carbon from the thioester linkage, breaking the bond and forming a glutamyl anhydride intermediate. The CoAS^−^ anion then attacks the carbonyl carbon of the glutamate, resulting in the covalent glutamyl-CoA thioester intermediate and the release of the donor carboxylic acid. In the second half of the reaction, the carboxyl oxygen of a suitable CoA-acceptor carboxylic acid attacks the carbonyl carbon of the glutamate, breaking the thioester intermediate and eventually yields a new CoA-derivative via a second anhydride intermediate (**Figure S1**).

To further characterize the *rip* operon on a genetic level, analysis of bacterial gene clusters containing all three *rip* genes suggest that there are other human pathogenic bacterial genera that also contain this operon, including *Salmonella enterica* [4]. Thus, the *rip* operon may function in a novel pathway required for bacterial replication in postactivated macrophages and may be important for virulence across distally related pathogens. In the first step to dissect this novel pathway on a molecular level, we show that *Y. pestis* RipA can bind a variety of CoA-derivatives. Further, CoA transferase activity assays suggest that RipA has a preference for butyryl or propionyl moieties compared to 4-hydroxybutyryl. Additionally, we report the 1.9 Å crystal structure of tetrameric RipA and demonstrate that this oligomeric state is stable in solution suggesting that the physiologically relevant assembly of RipA is tetrameric. Consistent with this, a molecular dynamics (MD) simulation shows that the tetramer is stable over the nanosecond time scale. Further analyses of the MD simulation reveal a variable active site pocket and provide insights into a possible gating mechanism for substrate binding. Finally, two hypotheses are discussed regarding the mechanism of action of how the *rip* operon may lower NO levels in macrophages. Together, our results provide a structural platform that may be used as a basis for rational inhibitor design against RipA.

## Results

### The rip operon is conserved in pathogenic bacteria

A protein BLAST search of available prokaryotic genomes reveals that the *rip* operon, which protects *Y. pestis* from NO macrophage assault [4], is also present in *Salmonella* and *Burkholderia* genera (**Table 1**). The identification of this novel operon in disparate pathogens suggests an evolutionarily conserved virulence pathway.

**Table 1:** The *rip* operon is conserved across distantly-related pathogens.

Genus Species (Serovar) Accession number	RipC	RipB	RipA
*Burkholderia mallei* NC_006349.2	46.3	74.9	59.9
*Burkholderia pseudomallei* NC_007435.1	46.3	74.9	60.1
*Salmonella enterica* (*Choleraesuis*) NC_006905.1	72.7	76.4	76.6
*Salmonella enterica* (*Typhimurium*) NC_003197.1	72.3	76.4	76.6
*Yersinia pestis* NC_004088.1	100	100	99.5
*Yersinia pseudotuberculosis* NC_006155.1	100	100	100

Bioinformatics analysis of Rip proteins performed on bacterial genomes available in the NCBI database. The table represents select pathogens harboring Rip homologs with a minimum of 45% amino acid identity and putatively organized in an operon (maximum intergenic distance of 1 kb). Percent identity between the *Y. pestis* Rip protein and the bacterial homolog is shown.

**Figure 1:**
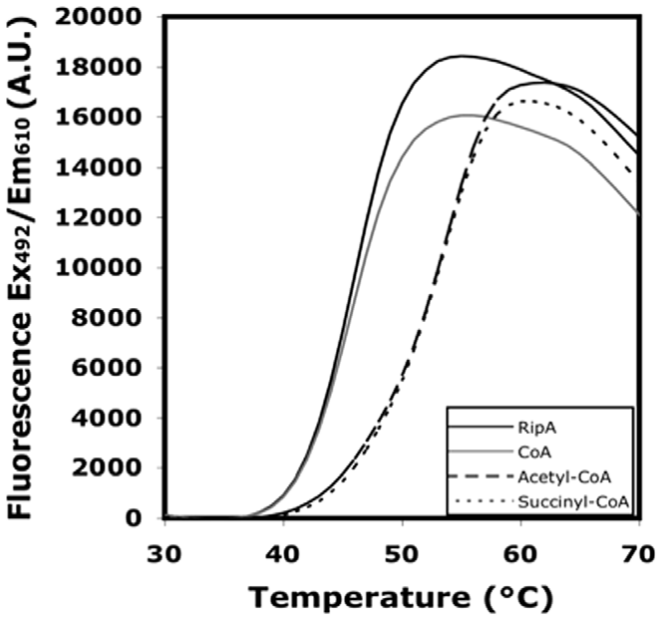
Analysis of RipA and its binding to CoA and CoA-derivatives. Representative DSF melting curves of 5 mM RipA incubated in the absence or presence of 20 mM CoA or CoA-derivatives at pH 7.4. Increased RipA thermostability is indicated by a rightward curve shift as seen for acetyl-CoA and succinyl-CoA. All experiments were at least performed in duplicate.

*RipA binding to CoA-derivatives is dictated by acyl chain length, branching and saturation*. To assess the ability of RipA binding to CoA-derivatives, we used Differential Scanning Fluorimetry (DSF), a rapid screening method that quantitatively measures thermal stability of protein-ligand interactions by observing changes in fluorescence intensity of SYPRO orange dye [12]. An interaction of a CoA-derivative with RipA should cause an increase in *T_m_*, which is indirectly measured by the increase of fluorescence as the dye binds to exposed hydrophobic regions during protein unfolding. RipA has a *T_m_* of 45.8°C in 0.1 M potassium phosphate buffer at pH 7.4 in the absence of substrates, as determined by the inflection point of the fitted sigmoidal curve (**Figure 1**). The addition of CoA to RipA did not affect the *T_m_* whereas CoA-derivatives (*i.e.*, acetyl-, butyryl-, succinyl- and propionyl-CoA) resulted in an increased *T_m_* between 7 - 7.7°C, indicating that an acyl moiety is necessary for CoA binding (**Figure 1**). The 10-carbon decanoyl-CoA and two branched CoA-derivatives, methylmalonyl-CoA and 3-hydroxybutyryl-CoA did not alter *T_m_* values while unsaturated crotonyl-CoA increased the *T_m_* slightly to 47.9°C (**Table 2**).

**Table 2:** DSF-derived *T_m_* values for RipA in the absence or presence of CoA and CoA-derivatives.

Sample	*T_m_* (°C)	SD
RipA	45.8	0.07
RipA + CoA	45.6	0.29
RipA + Acetyl-CoA	52.8	0.03
RipA + Butyryl-CoA	53.4	0.05
RipA + Succinyl-CoA	52.8	0.28
RipA + Propionyl-CoA	53.5	0.06
RipA + Crotonyl-CoA	47.9	0.54
RipA + Aceto-acetyl-CoA	48.3	0.01
RipA + Methylmalonyl-CoA	46.8	0.02
RipA + 3-hydroxybutyryl-CoA	46.1	0.17
RipA + Decanoyl-CoA	46.1	0.01

Each melting curve is fit by a nonlinear regression analysis using the Boltzmann function (GraphPad Prism) and the *T_m_* value is determined by the inflection point of the transition curve as defined from 35–65°C.

### CoA transferase activity assays

**Figure 2:**
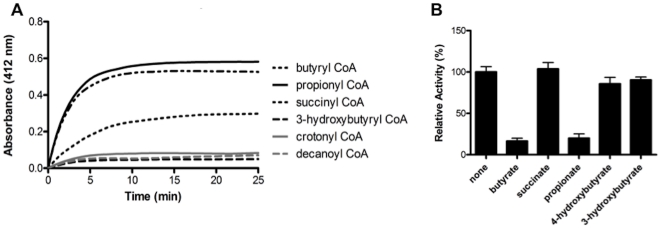
CoA transferase activity of RipA. (**A**) CoA transferase activity of RipA was measured with a variety of CoA-derivatives. Each 100 µL reaction mixture contained 50 µM CoA-derivative, 100 mM sodium acetate, 100 mM Tris pH 7.0, 1 mM oxaloacetate, 0.5 U citrate synthase and 1 mM DTNB. The reaction was initiated with the addition of 10 µM RipA and was incubated at room temperature for 30 min monitoring the release of free coenzyme A at 412 nm, which detects the formation of the nitrothiobenzoate dianion. CoA specificity was tested using different CoA donors with negative controls for background activity (without RipA) and CoA hydrolase (alternative CoA reaction). (**B**) Substrate specificity of RipA for the carboxylic acid. Acetate competed with a second acid in equimolar (10 mM each) in the presence of butyryl-CoA (100 mM). The remaining reaction mixture is the same as (**A**). The relative activities are compared to the reaction with no second carboxylic acid (labeled ‘none’).

The CoA transferase activity of RipA with various CoA-derivatives and acetate was determined using a coupled assay with citrate synthase assay monitoring the formation of free CoA [5]. In short, the rate of transfer of CoA from the donor CoA-derivative to acetate, catalyzed by RipA, to form acetyl-CoA was determined by observing the coupled release of CoASH from the condensation of oxaloacetate and acetyl-CoA by citrate synthase. The release of CoASH was detected by its reaction with 5,5′-dithiobis-(2-nitrobenzoic acid) (DTNB) at 412 nm. RipA CoA transferase activity was observed for butyryl- and propionyl-CoA and some activity for succinyl-CoA; however, little to no activity was observed with crotonyl-, decanoyl-, 3-hydroxybutyryl- (**Figure 2A**), methylmalonyl- and acetoacetyl-CoA (data not shown). RipA's promiscuity is not unprecedented, as other CoA transferases have been shown to non-specifically bind several CoA substrates [5], [10], [13], [14].

To test whether other acyl carboxylic acids can compete with acetate in the RipA catalyzed reaction of transfer of CoA from butyryl-CoA, a competition experiment was utilized, whereby a decrease in the rate of reaction suggests that a carboxylic acid is out competing acetate, thus resulting in a decreased rate of acetyl-CoA formation. 3-hydroxybutyrate and succinate do not compete with acetate while an 80% decrease in the rate of acetyl-CoA formation is observed with butyrate and propionate (**Figure 2B**). In contrast, only a 10% decrease in the rate of acetyl-CoA formation was observed with 4-hydroxybutyrate. These results reveal that butyryl and propionyl groups bind in favor of the acetyl moiety, suggesting that RipA may function as a butyryl- or propionyl-CoA transferase. Further analyses of the kinetics for RipA activity reveals that butyryl-CoA has a higher affinity than propionyl-CoA for RipA, although their rates of reaction are comparable (**Table 3**). This suggests that RipA has a preference for butyryl-CoA over propionyl-CoA.

**Table 3:** Kinetic constants for CoA-derivative substrates of RipA.

CoA-ester	*K_m_* (mM)	*k_cat_* (min^−1^)	*k_cat_*/*K_m_* (min^−1^mM^−1^)
Butyryl-CoA	188±20	2.97±0.25	1.58×10^−2^±0.15×10^−2^
Propionyl-CoA	281±27	3.11±0.23	1.10×10^−2^±0.11×10^−2^
Succinyl-CoA	1144±160	3.52±0.50	0.31×10^−2^±0.10×10^−2^

CoA transferase activity of RipA was measured with a variety of CoA-derivatives at varying concentrations. Each 100 µL reaction mixture contained CoA-derivative, 50 mM sodium acetate, 100 mM Tris pH 7.0, 1 mM oxaloacetate, 0.5 U citrate synthase and 1 mM DTNB. The reaction was initiated with the addition of 5 µM RipA and was incubated at room temperature for 30 min monitoring the release of free coenzyme A at 412 nm, which detects the formation of the nitrothiobenzoate dianion.

### Structural overview of RipA

All details of protein purification, crystallization and structure determination are in the Methods Section and **Table 4**. RipA was crystallized in the presence of CoA in space group P3_1_21, and acetyl-CoA and succinyl-CoA in space group C2, where both crystal forms contained two monomers per asymmetric unit. The RipA structure was solved by single-wavelength anomalous diffraction (SAD) of SeMet-derivatized RipA crystallized in the presence of acetyl-CoA, and molecular replacement was utilized to solve RipA crystallized in the presence succinyl-CoA and CoA, respectively. No density for CoA, acetyl-CoA or succinyl-CoA was observed in their respective models. We postulate that the low pH of the crystallization solutions may prevent ordered binding of CoA-derivatives, as no change in *T_m_* was observed in the presence of CoA-derivatives at pH 5.5, as determined by DSF (data not shown). The structural models of the RipA in the presence of CoA (3.0 Å) and RipA in the presence of acetyl-CoA (2.3 Å) were partially built whereby each RipA monomer was missing residues 337–345, whereas the model for RipA in the presence of succinyl-CoA (1.9 Å) was fully built. The structural model of RipA consists of a homodimer per asymmetric unit, where the RMSD between monomers is 0.20 Å for all atoms, and thus monomer A will be described in detail.

**Table 4:** X-ray diffraction data collection and atomic refinement for *Y. pestis* RipA crystallized in the presence of various CoA-derivatives.

	SeMet-RipA with Acetyl-CoA	Native RipA with Succinyl-CoA	Native RipA with CoA
Space Group	C2	C2	P3_1_21
Unit cell dimensions (Å)	117.5×109.3×85.3	118.5×108.5×84.9	197.5×197.5×61.1
pH of crystallization condition	5.7	5.0	6.3
Data set			
Wavelength (Å)	0.97930	1.0	1.0
Resolution range (Å)	50 – 2.3	50 - 1.9	50 - 3.0
Unique reflections (total)	38932 (411142)	71782 (239991)	27624 (404355)
Completeness (%)¶	94.7 (92.0)	98.10 (86.20)	100.00 (100.00)
Redundancy¶	10.5 (10.0)	3.3 (2.0)	14.6 (14.2)
R_merge_ ¶ ^,^ a	10 (54.3)	5.3 (15.9)	12 (56.1)
I/σ¶	9 (1.6)	19.7 (4.8)	28.0 (6.0)
FOM	0.30	-	-
# of Se sites	16	-	-
NCS copies	2	2	2
Model refinement			
Resolution range (Å)	26.7 - 2.3	41.23 - 1.90	49.72-3.0
No. of reflections (working/free)	38671/1947	70864/1974	26715/1922
No. of protein atoms	6660	7233	6660
No. of water molecules	160	419	-
No. of acetate molecules/monomer	-	1	-
Missing residues	337–345	-	337–345
R_work_/R_free_ b, %	20.9/25.2	17.2/20.3	18.4/21.7
R.m.s deviations			
Bond lengths (Å)	0.011	0.007	0.012
Bond angles (degrees)	1.53	1.07	1.62
Ramachandran Plot			
Most favorable region (%)	96	98	97
Additional allowed region (%)	3.77	2	2.88
Disallowed region	0.23	0	0.12
PDB ID Code	3S8D	3QLI	3QLK

¶ Statistics for the highest resolution shell are given in (brackets).

a R_merge_ = ∑|I−<I>|/∑I.

b R*_work_* = ∑|F_obs_−F_calc_|/∑F_obs_ R*_free_* was computed identically except where all reflections belong to a test set of 5% randomly selected data.

**Figure 3:**
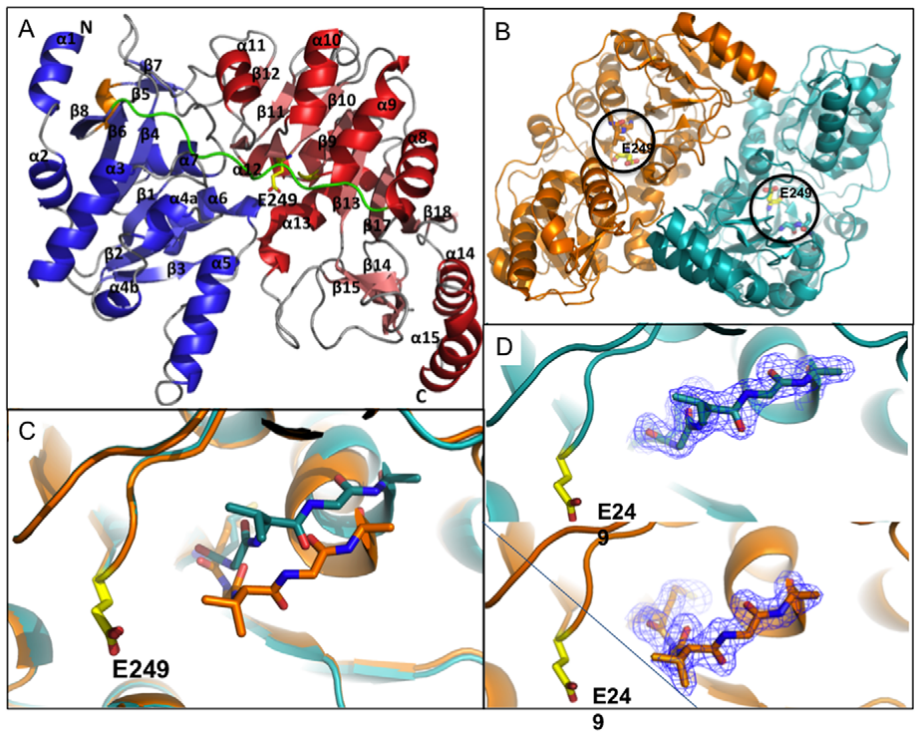
Structure of RipA. (**A**) Overall structure of *Y. pestis* RipA, the N-terminal and C-terminal domains are colored blue and red, respectively, connected via an ordered loop colored in green. The proposed active site glutamate, Glu249, and Val227, the key residue proposed to be involved in an active site “gating mechanism” are in stick representation with carbon, oxygen and nitrogen atoms are colored, yellow, red and blue, respectively. (**B**) Depiction of the tight dimer of RipA where each monomer of the dimer is colored in cyan and orange. The black circles indicate active site Glu249 and the three-residue loop (G-V227-G), which are in stick representation where oxygen and red atoms are colored red and blue, respectively, and Glu249 carbon atoms are colored yellow. (**C**) Superposition of the two monomers from the dimer, where the only difference between both subunits is in the three-residue loop (G-V227-G). (**D**) Electron density (shown in blue mesh) surrounding the GVG of the three-residue loop from each monomer of the dimer.

The RipA monomer consists of two open α/β domains, an N-terminal (1–190) and a C-terminal (204–440) domain connected via a polypeptide linker region (191–203) (**Figure 3A**). The N-terminal domain comprises of three layers in which a central six-stranded parallel β-sheet (β1, β2, β3, β4, β6, β8) is sandwiched between two additional layers. The outside layer consists of four α-helices (α2, α3, α4, α5). The inside layer consists of two α-helices (α6, α7) which interact with the C-terminal domain, and an α-helix (α1), a small 3_10_-helix and a two-stranded antiparallel β-sheet (β5, β7), which cap the end of the central layer b-sheet. The C-terminal domain has a similar three-layer topology, albeit its middle b-sheet layer is extended by two additional β-strands (β17, β18) to produce an eight-stranded mixed b-sheet. Furthermore, there is a short two-stranded parallel β-sheet near the domain interface, as well as an additional α-helix (α15) and β-turn near the C-terminus. The proposed catalytic glutamate, Glu249, is located in the C-terminal domain with the active site pocket extending from this domain interface toward the C-terminal α-helix (α15) (**Figure 3A**). Additional electron density near the active site glutamate suggests an acetate molecule is located within the active site (**Figure S2)**. Interestingly, although the RipA monomers are almost structurally identical, there is variability in a loop region adjacent to Glu249, where the three residue loop (Gly-Val227-Gly) has an alternate conformation (**Figures 3C and 3D**), where Val 227 protrudes 3.9 Å into the acyl-CoA binding cleft in monomer B, implications of which are discussed later.

**Figure 4:**
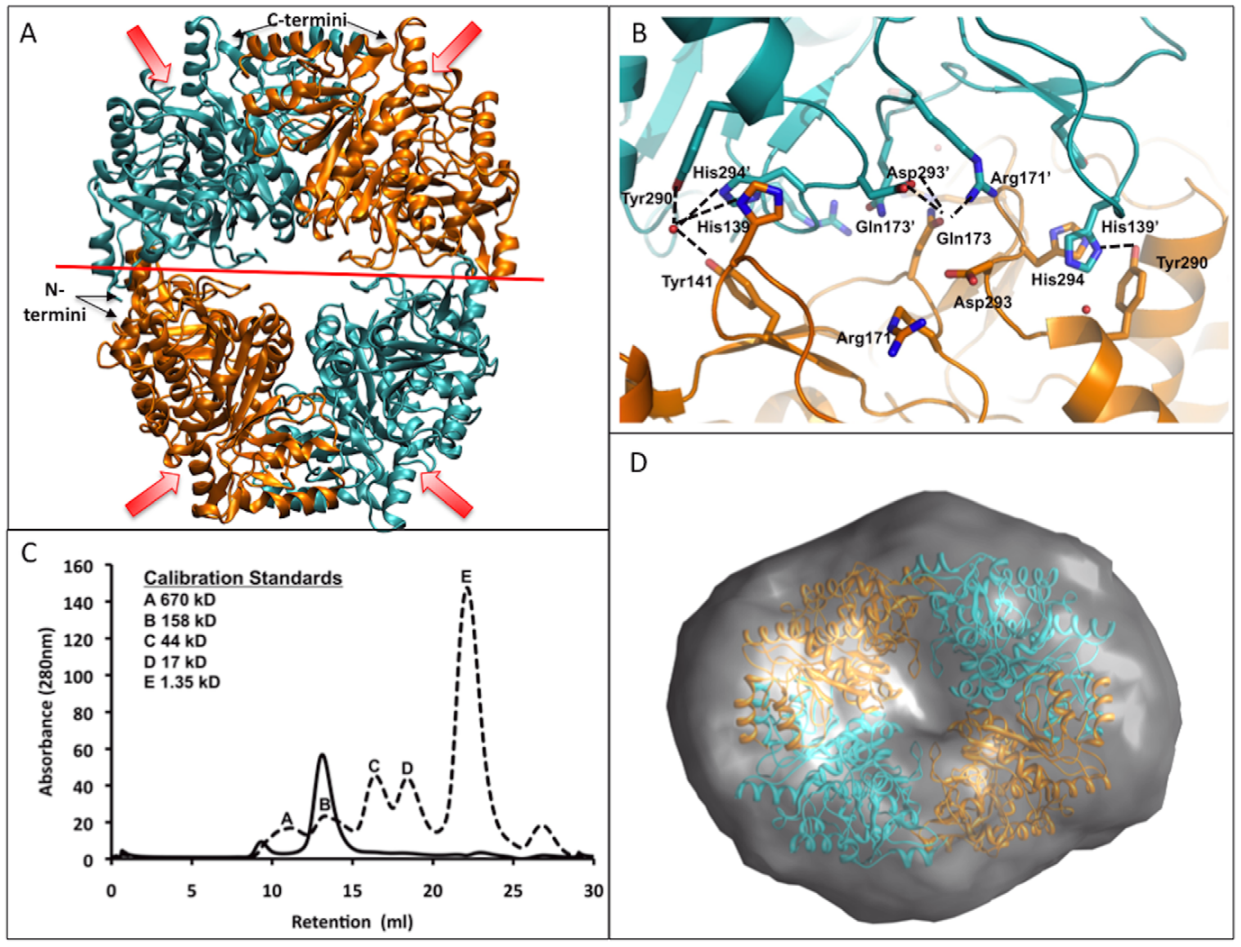
RipA is a tetramer. (**A**) Tetrameric state of RipA formed by the crystallographic symmetry axis at the dimer-dimer interface. Each monomer of the dimer are colored in cyan and orange, and the crystallographic symmetry axis is marked with a red line, and is at the dimer-dimer interface of the tetramer. Entrance to each active site of the four monomers is indicated with an arrow. (**B**) Hydrogen-bonding network at the dimer-dimer interface, one cyan and one orange monomer. Key interacting residues are in stick representation, where oxygen and nitrogen atoms are colored in red and blue, respectively. Black dashed lines indicate hydrogen bonds (less than 4 Å). (**C**) Size exclusion chromatogram of RipA. Bold line is RipA and dashed line is a protein standard. (**D**) Top view of SAXS data with the overlay of RipA tetramer with the electron-density envelope using CHIMERA. The envelope is calculated from an average of 10 GASBOR runs with P2 symmetry and 1756 residues.

Two RipA monomers form a dome-like homodimer in which the surface area of each monomer is ∼17700 Å^2^ and the buried surface area at the monomer-monomer interface is 1934 Å^2^ (calculated with AreaiMol [15]), ∼11% of the total monomeric surface area (**Figure 3B**). Residues forming the monomer-monomer interface are located in amino acid regions 99–123 of the N-terminal domain and 307–312, 347–357, 384–393, 411–406 and 438–439 of the C-terminal domain. The tight interaction at this interface is facilitated by eight residues from one monomer, which directly interact with the other monomer, where hydrogen bonds are less than 3.0 Å. Additionally, a network of water molecules facilitates various weaker interactions between the two monomers, as well as a pi-pi stacking interaction between His309 from both polypeptide chains. The homodimer is observed in crystal structures of 4-HB-CoATs from *Clostridium aminobutyricum* (3GK7) [14], *Shewanella oneidensis* (2OAS) and *Porphyromonas gingivalis* (3EH7). In comparison to other reported CoA transferases, a tetrameric state is observed through two-fold crystallographic symmetry (**Figure 4A**), a feature that is unique to *Y. pestis* RipA.

### RipA dimer-dimer interface

**Figure 5:**
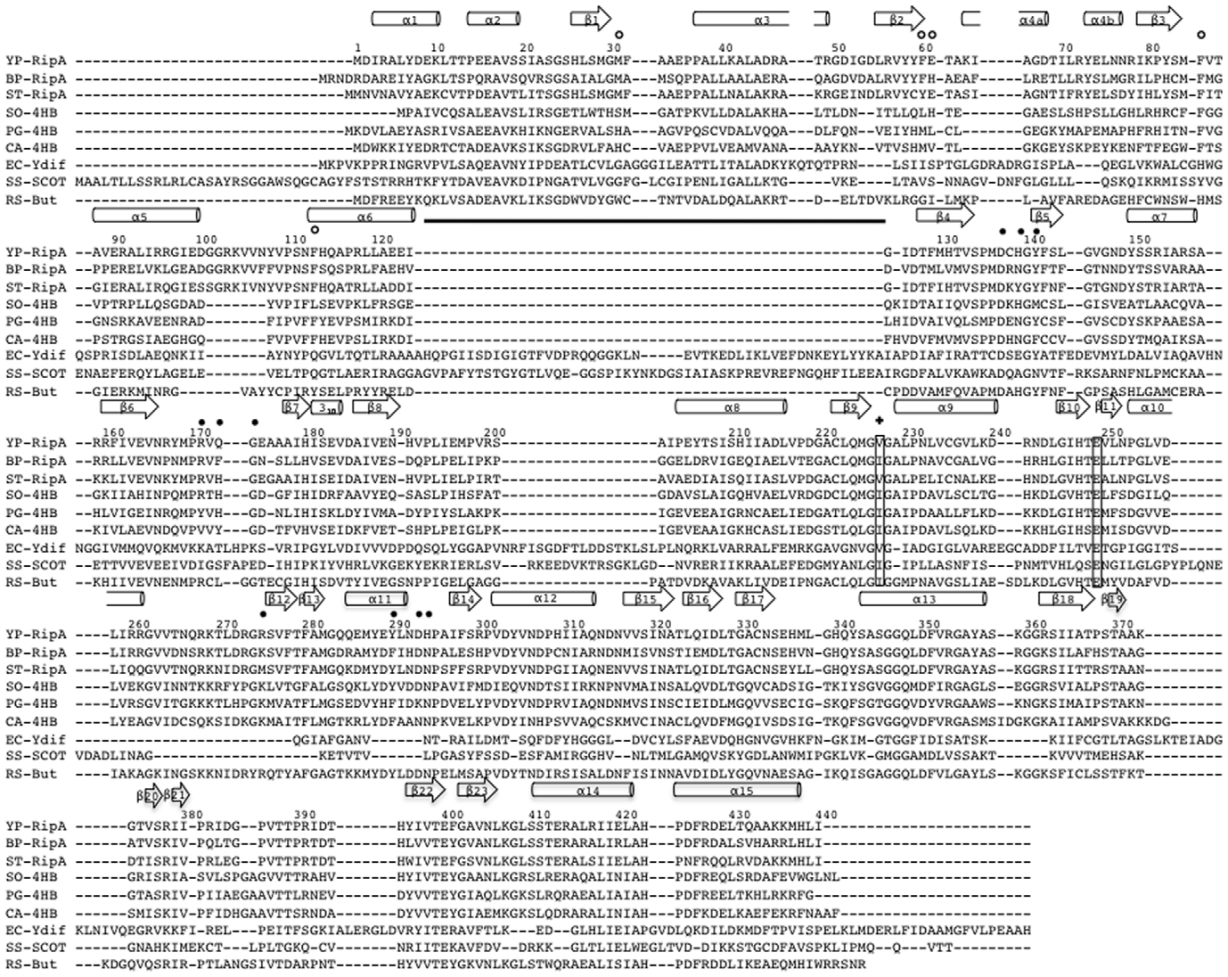
Sequence comparison of members of the Family I CoA transferases. Amino acid numbering and secondary structure elements above the sequence correspond to that of *Y. pestis* RipA, whereby tubes and arrows represent a-helices and b-strands, respectively. The conserved active site residue, Glu249, and the proposed key residue in the substrate gating-mechanism, Val227, are boxed. Residues donated with an open circle form the dimer-dimer interface, and those donated with a filled circle represent residues within the binding pocket of the acyl moiety for acyl-CoA. The sequence alignment of Family I CoA transferase homologs was made with ClustalW [55]. The black line represents the insert in *Escherichia coli* YdiF. Abbreviations of the species names for Family I CoA transferases are as follows: YP-RipA, *Yersinia pestis* RipA; BP-RipA, *Burkholderia mallei* RipA; ST-RipA, *Salmonella typhimurium* RipA; SO-4HB, *Shewanella oneidensis* 4-HB-CoAT; PG-4HB, *Porphyromonas gingivalis* 4-HB-CoAT; CA-4HB, *Clostridium aminobutyricum* 4-HB-CoAT; EC-YdiF, *Escherichia coli* YdiF; SS-SCOT, *Sus scrofa* SCOT; RS-But, *Roseburia* sp. Butyryl-CoA transferase.

Two homodimers form a doughnut-like tetramer where the buried surface area between each monomer at the dimer-dimer interface of the tetramer is 1454 Å^2^ (calculated with AreaiMol [15]), which is ∼8.2% of the total surface area. The dimer-dimer interface is facilitated by weak interactions between residues from both domains. Residues stabilizing the dimer-dimer interface include those from loop regions 137–141 and 173–175 of the N-terminal domain and 290–295 of the C-terminal domain, including Asp137, His139, Tyr141, Arg171, Gln173, Gly174, Arg275, Tyr290, Asp293 and His294 (**Figure 4B**), all of which are conserved across *Y. pestis*, *S. typumphium* and *B. mallei*, suggesting that the tetramer is specific to RipA homologs (**Figure 5**). The diameter of the tetramer is 106 Å×78 Å, and the central pore spans ∼8 Å at the minimum diameter. The residues lining the central pore are mainly polar residues, including Glu127, Arg153, Arg156, Arg268, Thr270, Arg273, Arg275, His309, Gln313, Asp315 and Lys359.

### RipA functions as a tetramer in solution

Two complementary methods were utilized to further confirm the oligomeric state of RipA in solution. Analytical gel filtration revealed that RipA eluted at a retention volume corresponding to ∼200 kDa (**Figure 4C**), consistent with a tetrameric state of RipA in solution (monomer 49 kDa, tetramer 196 kDa). Small angle X-ray scattering (SAXS) data further reinforced this result as the calculated molecular weight using SAXS MoW [16] corresponds to four molecules of RipA, and the electron density envelope fits the tetramer model from the structural model of RipA (**Figure 4D**). Gel filtration and SAXS data suggest that RipA forms a tetramer in solution and thus the tetrameric state observed in the RipA structural model is most likely physiologically relevant.

### Molecular dynamics of the RipA tetramer

It is commonly accepted that conformational flexibility and atomic-level dynamics play essential roles in facilitating protein function. Consequently, to add insight to the structural and experimental studies of RipA, we modeled the conformational dynamics of the RipA tetramer by performing a 20 ns of explicitly solvated molecular dynamics (MD) simulation under biologically relevant conditions. As a geometric measure of conformational diversity, alpha-carbon RMSD time series analyses were calculated for the tetramer and each monomer (**Figure S3**). The relatively small fluctuations for both the monomer and tetramer indicate stability. The time series of distances separating monomer centers of mass was determined as a metric of tetramer stability. In the crystal structure, each of these distances is identical, measuring 44.4 Å. During the simulation, these distances increase slightly, both converging to 45.2 Å within 1 ns (**Figure S4**). Relative to the rest of the monomer surface, the interface is slightly enriched in basic and polar residues and depleted of acidic and non-polar residues (**Table S1, Figure S5**). For each residue that spent at least 50% of the simulation within 5 Å of the adjacent monomer at the dimer-dimer interface, we identified interaction partners and calculated the fractional contact time spent with each (**Table S2**). These contacts, as well as a two-fold symmetry that exists at the dimer-dimer interface, are illustrated in **Figure 4B**. An ion pair between Arg171 and Asp293, not present in the crystal structure, forms across the dimer interface during the simulation. Additionally, residue His139, which is predicted to be doubly protonated, forms a charge-dipole interaction across the interface with the C-terminal of helix (α11). Stabilization of the interface is further enhanced with pi-pi stacking between His139 – His294 and hydrophobic interactions between Ala178 – Phe295.

### Investigation into the acyl-CoA binding pocket volume

**Figure 6:**
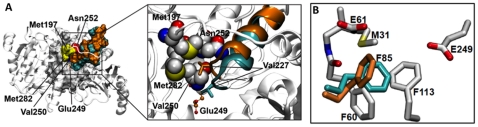
MD perturbations in the acyl-CoA binding pocket. (**A**) Val227 affects acyl-CoA binding pocket volume. In the overview (left), and the close-up (right) the representative small-volume conformation is shown in cyan, while the representative large-volume conformation is shown in orange. In the overview, molecular surfaces of residues within 10 Å are shown, while the remainder of the residues are rendered in cartoon. In the large-volume state, Val227 is stabilized by interactions with a pocket formed by the side chains of Val250, Met197, Met282 and Asn252; in the overview, these residues are rendered as colored surfaces and labeled (the methionines are colored yellow, asparagine is colored red and the valine is colored blue); in the close-up, these residues are rendered in van der Waals spheres and colored according to atom type. Glu249 is shown in orange spheres for reference. (**B**) Putative acyl binding pocket. Phe85 rotates during MD, increasing the available volume in a largely hydrophobic pocket adjacent to the putative catalytic Glu249. The position of Phe85 in the crystal structure is shown in cyan; a representative conformation from MD is shown in orange. Other residues lining the pocket that do not undergo substantial rearrangements during dynamics, along with Glu249, are shown in white. Pocket expansion likely facilitates acyl binding during the acyl transfer reaction.

The free volume of the acyl-CoA binding pocket was measured within 10 Å of the key catalytic residue, Glu249. The volume distribution revealed that the acyl-CoA binding pocket samples two distinct states (**Figure S6**): a “small-volume” state (65 Å^3^), which constitutes 6.3% of the sampled conformations, and a “large-volume” state (632 Å^3^), which makes up the remaining 93.7%. In all of the conformations from the small-volume state, a three-residue loop (Gly226, Val227, Gly228) connecting α9 to β9 protrudes into the active site. When the loop is extended, the side chain of Val227 juts into the CoA binding pocket, diminishing the free volume (**Figure 6A**, cyan). On the other hand, in the majority of the conformations from the large-volume state, the loop retracted, and the Val227 side chain is folded away from the CoA binding pocket and is sandwiched between the side chains of Met197, Met282, Asn252, and Val250 (**Figure 6A**, orange). Further, the Val227-loop extends and retracts multiple times during the 20 ns simulation. Notably, within the RipA crystal structure, the three-residue loop containing Val227 from each chain of the dimer adopt alternate conformations (**Figures 3C and 3D**) albeit less drastic than those of the MD simulation, reinforcing the suggestion of two volume states for RipA. Moreover, movement of Val227 into the CoA binding pocket constricts the passageway leading to the catalytic Glu249, which may hinder or occlude acyl-CoA binding (**Figure 6A** **, insert**).

Correlations between volume and the distance separating the a-carbon atom of Asn252 and the b-carbon atom of the Val227 side chain were investigated to determine the dynamic effects of Val227 loop extension on CoA binding pocket volume (**Figure S7**). Asn252 resides at the terminal of α10 and is adjacent to Val227 when the loop is retracted (**Figure 6A**). When Val227 extends into the active site, decreasing free pocket volume, it moves away from Asn252, and the distance increases. Consistent with this, the average distance in the small-volume state is 9.50+/−0.50 Å, while the distance in the large-volume state is 7.36+/−1.6 Å.

### Dynamics of the putative acyl-binding pocket

RipA demonstrates some degree of acyl-CoA promiscuity (**Figures 1** **and** **2**, **Table 2**), as observed in other Family I CoA transferase members [10], [14]. These observations suggest the existence of an acyl-binding pocket adjacent to the catalytic Glu249, which may determine acyl transfer specificity.

The MD trajectory reveals an acyl-binding pocket located on the N-terminal-domain, opening toward Glu249 (**Figure 6B**). Comprised of a triad of phenylalanines (Phe60, Phe85, and Phe113), the pocket is largely hydrophobic. Met31 and Glu61 are adjacent to the phenylalanine triad. Using the program PROPKA [17] during simulation preparation, the p*K*a of Glu61 was predicted to be 7.30. This value is consistent with the large unfavorable free energy change expected upon burying a negatively charged carboxylate in a hydrophobic pocket. Therefore, the simulations were carried out with Glu61 protonated, making it the sole hydrogen bond donor or acceptor. Notably, the pocket is only partially formed in the crystal structure, where the Phe85 side chain is pointing back toward Phe113 (**Figure 6B**), creating a shallower pocket than that observed during the MD simulation. During simulation equilibration, the Phe85 side chain rotates away from its position in the crystal structure, increasing pocket depth (**Figure 6B**). After rearranging into the new conformation, Phe85 is stable over the course of the MD simulation. The side chain of Glu61 also rotates, mediating the position of the carboxylic acid relative to the phenylalanine triad and populating three states (**Figure S8**). In the most and least populated states, the carboxylic acid is rotated downward and is positioned directly above the phenylalanine triad, where it may serve as a hydrogen bond donor or acceptor for charged or polar acyl groups, such as succinate. In the second most populated state, the carboxylic group is positioned upward, away from the phenylalanines, modestly expanding the pocket. Phe85, Phe113 and Met31 are stable and fluctuate little compared to the motion observed in Glu61.

**Figure 7:**
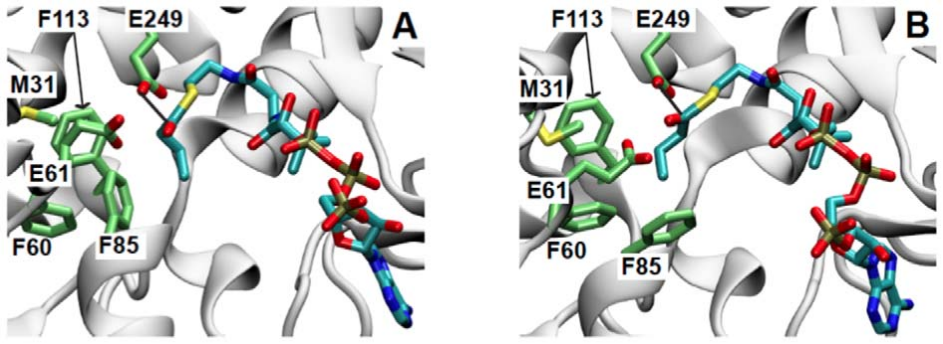
Modeling butyryl-CoA into the RipA structure. (**A**) Butyryl-CoA was modeled into chain A of the crystal structure. Phe85 closes the predicted acyl-binding pocket, forcing the butyryl to bend back toward the AMP-CoA moiety. The distance between the Glu249 putative nucleophile and the carbon atom of the carbonyl elecrophile is 4.95 Å. (**B**) Butyryl-CoA was modeled into a conformation sampled from the MD simulation. Phe85 rotates, opening the predicted acyl-binding pocket and allowing the butyryl to adopt a more extended conformation. In the more extended conformation, the distance between Glu249 and the carbonyl electrophile closes to 3.74 Å. In both (A) and (B) protein residues are colored by atom type with green carbon atoms, while butyryl-CoA is colored by atom type with cyan carbon atoms; residues not shown explicitly are rendered in white cartoon.

To further explore the possible functional significance of the expanded acyl-binding pocket and protonation state of Glu61, we modeled butyryl-CoA into the crystal structure and an open-pocket conformation from the MD simulation. The butyryl groups were added to the CoA conformation taken from the *S. oneidensis* 4-HB-CoAT CoA-bound structure and manually positioned to minimize steric conflict with the protein. Each butyryl-CoA was then briefly minimized while fixing the position of the protein, resulting in the final models (**Figure 7**). In the crystal structure model, Phe85 prevents the butyryl group from binding in the predicted acyl-binding pocket (**Figure 7A**). This forces the butyryl group to fold back toward the solvent, pushing the thioester carbonyl group away from the putative Glu249 nucleophile. In the MD-model, the open acyl-binding pocket clearly accommodates the butyryl group (**Figure 7B**), which permits a more extended butyryl conformation and allows the carbonyl thioester and Glu249 to move roughly 1 Å closer to one another. In both models, Glu61 is positioned to donate a hydrogen bond to the thioester carbonyl oxygen atom.

### Ensemble-averaged electrostatics of RipA

**Figure 8:**
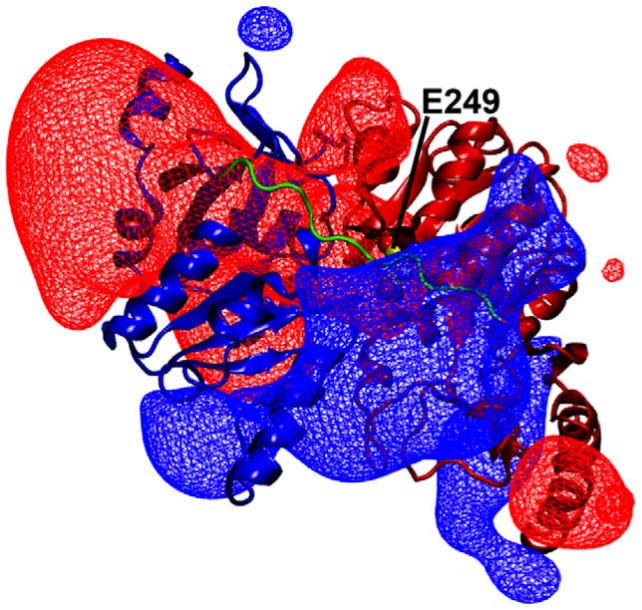
Ensemble averaged electrostatics. Positive and negative electric isopotential surfaces, with values +70 kT/e and −70 kT/e, respectively, are shown in blue and red wire frame mesh. The N-terminal domain is colored blue, the C-terminal domain red, and linker green. For reference, Glu249 is shown in yellow van der Waals representation and labeled, and RipA is oriented as in Figure 3A.

Ensemble-averaged electrostatic potentials have been shown to agree better with experimental data for a number of biologically relevant systems [18]. To gain further insight into the physical factors that may influence the structure and function of RipA, we calculated the enzyme electrostatic potential, averaged over the four monomers and the ensemble of conformations generated during the 20 ns MD simulation. A large positive electric isopotential surface (+70 kT/e) is positioned directly above the acyl-CoA binding cleft and extends roughly 10 Å beyond the protein-solvent interface into bulk solvent. Similarly, large negative electric isopotential surfaces (−70 kT/e) are positioned on either side of the acyl-CoA binding cleft (**Figure 8**).

## Discussion

### The conserved rip operon may constitute a novel virulence pathway

The *rip* operon is observed across *Yersinia*, *Salmonella* and *Burkholderia* species (**Table 1**). To further support the hypothesis that RipA, RipB and RipC are important in bacterial survival for this diverse set of pathogens, studies in *Salmonella typhimurium* (serovar of *S. enterica*) revealed that the *ripA* and *ripB* genes are important virulence factors which are upregulated in *S. typhimurium*-infected macrophages [19], and are further required for *S. typhimurium* mouse systemic infection [20], [21]. The conserved *rip* operon appears to be required for infection and virulence in *Salomonella*, as well as *Yersinia* virulence, and thus may constitute a novel virulence pathway.

The gene products from the *rip* operon are highly homologous to proteins involved in the production of CoA-derivatives. The closest amino acid sequence homologs to RipA, RipB and RipC are 4-HB-CoAT, enoyl-CoA dehydratase/hydratase and citrate lyase β-subunit, respectively. Significantly, citrate lyase β-subunit is involved in the reversible conversion of citryl-CoA to acetyl-CoA [22]. Thus, it is highly plausible that the *rip* operon is involved in the production of a CoA-derivative and an acyl carboxylic acid.

### RipA is a Family I CoA transferase

RipA belongs to the Family I CoA transferases (**Figure 5**), which consists of a wide variety of proteins found both in eukaryotic and bacterial species and is characterized by two main features: an active site glutamate, Glu249, essential for the ping-pong mechanism of CoA transferases and a similar α/β fold [6]. Sequence alignment of RipA to other Family I CoA transferases reveal the conserved catalytic glutamate, as well as high sequence identities to *Roseburia* sp. butyryl-CoA transferase (39%) ([13]) and 4-HB-CoATs from *Clostridium aminobutyricum* (38%, PDB:3GK7) [14], *Porphyromonas gingivalis* (40%, PDB:3EH7) and *Shewanella oneidensis* (40%, PDB:2OAS) (**Figure 5**).

As there are no existing butyryl-CoA transferase structures, the 1.9 Å RipA crystal structure, which demonstrates the canonical Family I CoA transferases α/β fold, is compared against 4-HB-CoAT structures. The overall fold amongst the RipA and 4-HB-CoAT structures are highly conserved with RMSDs between 1.57–1.68 Å in pairwise structural alignments. In the *S. oneidensis* 4-HB-CoAT structure, helix α1, which is conserved in RipA and the other 4-HB-CoAT structures, is missing. However, the most distinct difference between RipA and the other 4-HB-CoATs is the extension of helix α5 (**Figure 3A**), the consequence of which induces a variety of structural differences that are described below. Firstly, α5 pivots 30° at the N-terminal end away from the surface of the molecule, preventing steric clashes with α15 from the adjacent monomer. Concurrently, the N-terminal end residues of α5 protrudes toward the β15 to α13 loop, which sits along the proposed CoA binding pocket, causing a 90° rotation of the loop. This rotated loop, in turn, induces the formation of an extended kinked helix (α13) at its base, at the expense of a β-turn found in other 4-HB-CoAT structures [14] and forms unique contacts with a truncated loop between β16 and β17.

The differences between the 4-HB-CoATs and other Family I CoA transferases, including SCOT and YdiF, consist of several insertions as previously described [14]. In addition, unlike in the other Family I CoA transferases, 4-HB-CoATs have an ordered linker that crosses adjacent to the CoA active site. Consistent with the high degree of monomeric structural conservation among the 4-HB-CoATs, the monomer-monomer interfaces are similar with variations isolated to interactions between non-conserved residues while more distinct differences are observed between other CoA transferases.

### RipA has a unique tetrameric orientation

The tetrameric structure of RipA is unique compared to the previously described *E. coli* YdiF (PDB Code: 2HAU) [10], which is further validated as the YdiF tetramer assembly does not fit into the RipA SAXS electron density envelope. The reported YdiF structure has a dimer-dimer interface with weak N-terminal domain interactions, resulting in only ∼2% total buried surface area for each monomer (**Figure S9**). This interface is stabilized by two direct protein contacts and a network of ordered waters, whereby one dimer appears to float on top of the other (**Figure S9**). In contrast, the RipA dimer-dimer interface has far more extensive residue contacts from both domains and each monomer has a total buried surface area of ∼8.2%. Although a network of ordered dimer-dimer interface waters facilitates tetramer formation in both RipA and YdiF, the increased buried surface and direct residue contacts found in RipA likely results in a more stable tetramer. The difference in tetrameric structures appears to originate at the dimer level. Compared to the other 4-HB-CoAT structures found in the PDB, YdiF has a unique insertion between α6 and β4 (**Figure 5**). This insert, which consists of five antiparallel β-strands and one α-helix (**Figure 5**), leads to the noted differences at the dimer interface. On the other hand, the CoA bound *S. oneidensis* 4-HB-CoAT (PDB:2OAS, unpublished) tetramer is similar to that of RipA. Like the RipA tetramer, it is formed through crystallographic symmetry and has a conserved dimer-dimer interface. Conservation includes residues Asp137, Tyr141, Arg171, Gly174, and Asp293, all of which are found in RipA homologs and *Roseburia* butyryl-CoA transferase (**Figures 4B** **and** **5**). This unique RipA tetrameric state appears stable both experimentally and during molecular dynamics simulations suggesting a possible role in RipA function.

### RipA is a probable butyryl-CoA transferase

The functional annotations of CoA transferases are determined from the identities of cognate acyl-CoA substrates. However, non-cognate CoA-derivatives have also been reported to interact with several CoA transferases at varying specificities, suggesting substrate promiscuity [7], [23], [24]. As such, RipA shows similar reactivity with multiple acyl-CoA substrates as suggested from DSF and shown by activity assay results (**Figures 1** **and** **2** **,** **Table 2**). These results do show a profile corresponding to a role in short-chain, unbranched and unsaturated CoA-derivatives binding. Intriguingly, the RipA CoA transfer activity rate is fastest in the presence of butyryl- and propionyl-CoA (**Figure 2A**). Moreover, butyrate and propionate are preferred to acetate in competition assays (**Figure 2B**), which is similar to substrate specificity previously observed in *Roseburia* butyryl-CoA transferase [13]. Conversely, in competition assays with 4-HB-CoAT from *C. aminobutyricum*, 4-hydroxybutyrate greatly out-competes butyrate and propionate, both of which are preferred to acetate [5], [13]. Furthermore, kinetic analysis confirms that butyryl-CoA is the preferred substrate over propionyl-CoA (**Table 3**). Thus, the previously annotated CoA transferases together with our experimental results suggest that RipA is a butyryl-CoA transferase.

### Insights into substrate binding and specificity

As no Family I CoA transferase structures bound to their acyl-CoA substrates exist, the mechanism for determining specificity is not explicitly known. We speculate that the fluctuating putative acyl-binding pocket, as revealed in the MD simulations, may play a role in substrate specificity.

To facilitate catalysis, the acyl-CoA carbonyl must be proximal to the putative catalytic Glu249. This requirement likely forces the acyl backbone into the adjacent N-terminal pocket formed by Phe85, Phe60, Phe113, Met31 and Glu61 (**Figures 6B** **and** **7**). With the exception of Glu61, which is protonated and polar, the pocket is entirely apolar, resembling those described in *C. aminobutyricum* 4-HBCoAT [14] and SCOT [25], [26] with Phe85, Phe60 and Phe113 conserved in all 4-HBCoATs. The pocket size, which restricts the size of the acyl group that can bind effectively, appears to be large enough to accommodate acyl groups of three to four carbon atoms and helps explain the competition and DSF assays. To illustrate this, we modeled butyryl-CoA into the crystal structure and an open-pocket conformation from the MD simulation (**Figure 7B**). The crystal structure occludes butyryl, which shifts the carbonyl thioester electrophile away from the Glu249 nucleophile, to a distance of 4.95 Å (**Figures 7A & 7B**). In contrast, the open acyl-binding pocket is able to accommodate the butyryl group, reducing the distance between electrophile and nucleophile to 3.74 Å. Thus, our models suggest that the open pocket may influence the orientation of the thioester and facilitate reaction. The models also predict that acyl groups longer than four carbon atoms are too large for even the open pocket (**Figure 7A**), in support of the DSF and activity data for decanoyl-CoA (**Figures 1** **and** **2** **,** **Table 2**). Furthermore, our models indicate that the apolar pocket will interact favorably with hydrophobic molecules, such as propionyl and butyryl. Similarly, we hypothesize that the apolarity of the acyl-binding pocket is insufficient to orient succinate in a reactive conformation, as suggested by the low affinity of RipA for succinyl-CoA (**Table 3**). This is also consistent with competition assay results for 3-hydroxybutyrate and 4-hydroxybutyrate.

Notably, the position of the Met31, conserved only within *rip* operon RipA homologs (**Figure 5**), suggests that RipA has a different acyl-CoA specificity than 4-HBCoATs; the hydrophobic side chain will sterically clash with the proposed hydrogen bonding between the corresponding 4-HBCoAT His31 and the 4-hydroxy group of the 4-hydroxybutyrate-CoA substrate. These observations suggest a possible unique functional role for RipA, in which specificity for the acyl-CoA substrate is controlled by the phenylalanine triad pocket; and together with the functional analysis, the data suggest that RipA is predominately a butyryl-CoA transferase.

### Potential mechanism of substrate association for Family I CoA transferases

As Family I CoA transferases have conserved active sites, the mode of substrate binding that can be gleaned from RipA MD simulations may describe a generalized mechanism for other members of the family. To progress through the catalytic cycle, RipA must first bind an acyl-CoA substrate. The electric field around RipA may facilitate binding by channeling the acyl-CoA thioester, which, assuming a neutral acyl group, has an overall −4 charge, toward the active site. The electric isopotential surface is prominently positioned above the active site, where it can attract the negatively charged acyl-CoA into the binding cleft (**Figure 8**).

When comparing the two monomers within the dimer of the RipA crystal structure, it was found that monomer B has a loop containing Val227 that moves into the CoA pocket (**Figures 3C and 3D**). This movement is similar to that observed in recent unbound and CoA-enzyme intermediate forms of SCOT that show that the C-terminal domain moves toward the N-terminal domain, constricting the acyl-CoA binding pocket in the CoA-enzyme intermediate state [25], [27]. The RipA structure most likely represents a form similar to the unbound form crystallized with glycerol [27], as RipA was also crystallized with glycerol. In particular, the authors note that movement of the Val227 homolog, Ile284, toward the catalytic glutamate may aid in shielding the thioester from hydrolysis; it is plausible that Val227 plays a similar role in RipA.

In the RipA simulations, the Val227-loop also moves in and out of the CoA binding cleft, and this motion is correlated with CoA binding cleft volume changes (**Figure 6A** **, Figures S6 and S7**). This suggests that once the acyl-CoA encounters RipA, Val227-loop dynamics may “gate” the CoA binding pocket and affect the rate of acyl-CoA binding. The phenomenon of active site gating has been described in a number of systems [28]. In order to determine whether this gating affects the acyl-CoA binding rate, the average dissociation time of the encounter complex, as well as the gating period (the average time required for Val227-loop extension and retraction) need to be compared. The Val227-loop extends and retracts multiple times during our 20 ns simulation, indicating a gating period on the order of nanoseconds. A rough approximation of the average dissociation time of the encounter complex using diffusion constants estimated with the Stokes-Einstein equation gives a value of 0.12 µs. This rough analysis indicates the gate opens and closes ∼100 times before the encounter complex dissociates, and it is unlikely that gating motion slows the overall acyl-CoA association rate.

In addition, the transient constriction of the acyl-CoA binding pocket may be indicative of a conformational state that assists catalysis upon acyl-CoA binding. The idea that enzymes transiently populate, or “pre-select” conformational states that assist catalysis, even in the absence of substrate, has been observed in a number of enzymes [29], [30], [31]. Although we currently lack a crystal structure of CoA bound to RipA, the Val227-loop conformations sampled during MD in context of the recent SCOT structures [25] suggest that RipA follows a conformational pre-selection mechanism. Together, conformational pre-selection and the gating analysis suggest that the Val227-loop structure is highly evolved to optimize catalytic efficiency, sampling conformations relevant to catalysis without adversely impacting the rate of acyl-CoA association.

### Biological implications

It was previously suggested in *S. typhimurium* studies that the Rip proteins play a role in the synthesis of peptidoglycan, which have a vital role in infection [19]. However, in *Y. pestis* the *rip* operon has been implicated in enhancing intracellular viability by lowering the level of NO in postactivated macrophages [4], suggesting that either one or all of the gene products may be involved in direct or indirect NO detoxification. We propose two possible hypotheses whereby RipA produces either: (i) acetyl-CoA that may bind to and detoxify NO, yielding S-nitrosoCoA [32], [33], or (ii) butyrate, a known anti-inflammatory, which has been shown to reduce NO levels in INF-g activated macrophages [34], [35]. Collectively, the results presented here favor the latter hypothesis, in which RipA is a butyryl-CoA transferase.

In conclusion, to dissect the role of this novel *rip*-mediated pathogenesis pathway, we have focused our efforts on the biochemical, structural and computational characterization of *Y. pestis* RipA. Herein, we suggest that in INF-g activated macrophages, RipA, a proposed butyryl-CoA transferase, produces butyrate that indirectly lowers the macrophage NO levels, preventing bacterial cell death. Furthermore, we now have a structural platform for rational inhibitor discovery to probe this pathway with small molecules *in vitro* and to shed light on the mechanism of action of the encoded *rip* operon.

## Materials and Methods

### Overexpresion and purification of RipA

RipA was overexpressed in a pET28 plasmid containing the *Y. pestis ripA* gene using *E. coli* BL21(DE3) Gold cells (Strategene). Cells were grown at 37°C in LB medium containing 50 µg/ml of kanamycin. Protein expression was induced by adding 1 mM IPTG at an OD_600_∼1.0 and grown at 18°C overnight before harvesting. Cells were pelleted at 5,000 rpm for 10 min and then resuspended in wash buffer (50 mM Tris pH 7.4, 350 mM NaCl, 10 mM imidazole and 10% glycerol) containing phenylmethylsulfonyl fluoride (PMSF) and hen egg lysozyme. Then cells were lysed by sonication and centrifuged at 13,000 rpm for 40 min followed by filtration (0.22 mm) of the cell lysate to remove cell debris before purification. The cell lysate was loaded on to a Ni^2+^-charged HiTrap column (5 mL) and washed with wash buffer before protein was eluted with an imidazole 10–500 mM linear gradient (100 ml) in which purified protein eluted between 200 and 300 mM imidazole. The fractions were collected and concentrated in a centricon (15 mL) to 6 mg/ml. The selenomethionine-derivatized RipA was grown in M9 minimal medium supplemented with amino acids supplements (leucine, isoleucine, valine, 50 mg/L; phenylalanine, lysine, threonine, 100 mg/L; and selenomethionine 75 mg/L) adapted from a previously described protocol [36]. The SeMet-RipA was purified as described for the native RipA above.

### Differential scanning fluorimetry (DSF)

The thermal stability of RipA with various CoA-derivatives was assessed using an Mx3005P QPCR machine (Agilent). Each 50 mL sample contains 5 µM RipA in 100 mM potassium phosphate buffer pH 7.4, incubated with 20 µM CoA-derivative in the presence of 40 µM SYPRO orange dye. Fluorescence readings (Ex/Em wavelengths: 492/610 nm) were recorded from 25–95°C with a temperature ramp of 1°C/min [12]. All samples were tested in duplicate and the results duplicated in at least two independent assays. The data were fitted by a nonlinear regression analysis using the Boltzmann function (GraphPad Prism) and *T_m_* values were determined by the inflection point of the resulting transition curves as defined from 35–70°C.

### CoA transferase activity assay

RipA enzymatic activity assays was carried out as previously described [5], whereby transfer of CoA from a CoA-derivative (Sigma) to acetate was determined by the coupled release of CoASH from the condensation of oxaloacetate by citrate synthase (Sigma) and detected at 412 nm by 5,5′-dithiobis-(2-nitrobenzoic acid) - DTNB. Each 100 µl reaction mixture contained 50 µM CoA-derivative, 100 mM sodium acetate, 100 mM Tris pH 7.0, 1 mM oxaloacetate, 0.5 U citrate synthase and 1 mM DTNB. Reactions were initiated with the addition of 10 µM RipA and monitored for 30 min at 412 nm at room temperature, detecting the formation of the nitrothiobenzoate dianion. The negative control was the same reaction mixture without citrate synthase. Competition assays were performed as above, however equimolar amounts of sodium acetate (10 mM) and another carboxylic acid where added to the reaction mixture to observe if RipA favors the new carboxylic acid as a substrate over acetate by the inhibition of acetyl-CoA production, whereby RipA favors the new carboxylic acid as a substrate over acetate. Kinetic experiments were carried out for RipA (5 µM), in which the concentrations of succinyl-, butyryl- and propionyl-CoA were varied. All experiments were performed in at least three independent assays.

### Sodium 4-Hydroxybutyrate Synthesis

Sodium 4-hydroxybutyrate was prepared from an adapted protocol as previously described [37]. Briefly, 16.3 g of 4–butyrolactone (Sigma) and 7.4 g of sodium hydroxide was initially dissolved in 30 mL of water and then refluxed for three hours. Additional water was added to fully dissolve the salt and the resulting solution was filtered and evaporated to dryness under reduced pressure. Sodium 4-hydroxybutyrate was recrystallized from ethanol and its purity was confirmed by nuclear magnetic resonance.

### Crystallization of RipA

RipA crystallized in two different space groups, C2 and P3_1_21. Protein concentrations for crystallization are given in **Table 4**. Native and SeMet-derivatized crystals in space group C2 were grown over a period of one week at room temperature by hanging drop-vapor diffusion with a reservoir containing 8% tacsimate pH 5.0, 8% PEG 3350, 4% v/v 2,5-hexandiol and 1 mM acyl-CoA (succinyl- or acetyl-CoA, respectively) with crystallization drops on a 1 µL∶1 µL protein to reservoir. Crystals in space group P3_1_21 were grown similarly in 18% PEG 3350, 0.2 M sodium citrate, 0.1 M Bis-tris propane, pH 6.3 and 1 mM CoA. Crystals were mounted and collected under cryoconditions with the addition of 25% glycerol as cryoprotectant to the reservoir condition.

### Data collection, structure determination, and refinement

RipA crystallized in two different space groups, C2 and P3_1_21, where both space groups contain two monomers per asymmetric unit. A Se-single anomalous dispersion (SAD) dataset at the Se absorption edge (0.9793 Å) was collected from the C2 form on beamline 5.0.2 at the Advanced Light Source (ALS). Data reduction was carried out with the HKL2000 suite [38], resulting in a 98% complete dataset up to 2.3 Å, with significant anomalous differences up to 3.6 Å resolution. Phenix.autosol [39] located 16 Se sites and identified a two-fold non-crystallographic symmetry operator. Subsequent density modification resulted in electron density maps that were subjected to automated and manual model building procedures. The final model was built through iterative building in Coot [40] and refining through Phenix.refine [39]. The C2 crystal form crystallized in the presence of succinyl-CoA was collected similarly to 1.9 Å with final R_work_/R_free_ (%) 17.2/20.3 with 98% in favorably allowed regions. The P3_1_21 crystal form crystallized in the presence of CoA was collected similarly to 3.0 Å, and solved by molecular replacement utilizing Phenix [39]. Programs from the CCP4 package [41] as well as Phenix [39], Pymol [42], and Coot [40] were used to analyze the stereochemistry and geometry of the models and were found to be acceptable. Data collection and refinement statistics are presented in **Table 4**.

### Gel Filtration

Tertiary structure was determined by analytical gel filtration where 500 ml of protein was run on a Superdex 200 using an AKTA FPLC and compared to a protein standard (Biorad).

### SAXS analysis

SAXS was used for similar results with 3 different measurements at different concentrations with resultant data processed through PRIMUS and GNOM [43]. Resultant tertiary structure was determined both from calculated molecular weight from SAXS MoW [16] and fitting of the RipA tetramer in a SAXS envelope generated from GASBOR using 10 simulations using P2 symmetry averaged together with DAMAVER [43] and the envelope generated using CHIMERA [44].

### Molecular Dynamics system preparation

Protein parameters were assigned according to the AMBER 99ffSB force field [45]. Protonation states at a pH of 7.4 were predicted using the program PROPKA, and hydrogen atoms were assigned according to residue templates in the AMBER 99ff using the xleap program accompanying the AMBER 10 suite [46]. The initial CoA-tetramer model was immersed in a TIP3P [47] solvent box that provided a 10 Å buffer between the protein and the boundary of the periodic box in each direction. Sodium ions were added to bring the system to electric neutrality. Additional sodium and chloride ions were added to bring the sodium chloride concentration to 20 mM.

### Molecular dynamics simulations

Following model construction and parameterization, 10 kcal mol^−1^ Å^−2^ harmonic restraints were applied to the backbone and 5 kcal mol^−1^ Å^−2^ harmonic restraints were applied to the side chains. The model was then minimized in 5 stages. A restraint-scaling factor was applied and reduced linearly from 1 to 0.25 in 0.25 increments over 4, 5000-step minimization stages. During the final stage, the restraints were removed, and minimization was performed for 10,000 steps. Following minimization, equilibration was carried out at 1 atmosphere and 300 Kelvin (NPT ensemble). The temperature was maintained using Langevin dynamics with a damping coefficient of 5 ps^−1^, while the pressure was maintained using a hybrid Nose-Hoover-Langevin piston method [48] with period and decay times of 200 and 50 fs, respectively. A multi-time step algorithm was used in which bonded interactions were computed every time step, short-range nonbonded interactions were calculated every 2 time steps, and full electrostatics were computed every 4 time steps. The water hydrogen-hydrogen and hydrogen-oxygen distances were constrained using the SHAKEH algorithm [49] to be within 0.0005 Å of the nominal force-field length. The particle mesh Ewald method [50] was used to treat long-range electrostatics, using a grid spacing of 1.0 Å. An integration time step of 1.0 fs was used. During equilibration, 10 kcal mol^−1^ Å^−2^ harmonic restraints were applied to the backbone, and 5 kcal mol^−1^ Å^−2^ restraints were applied to the side chains. A restraint-scaling factor was used and reduced linearly from 1 to 0 in 0.25 increments in 5 stages, each lasting 250 ps. Following equilibration, production MD of the tetramer model was carried out for 20 ns, sampling conformations every 50 ps, for a total of 40,000 model conformations. Production MD parameters were identical to those used during equilibration, but no restraints were applied. Minimization, equilibration, and production MD were all carried out using NAMD2.7 [51]. Production MD was performed on the TeraGrid Ranger cluster, using 560 processors. A typical benchmark on the ∼200,000 atom system was 0.135 days per nanosecond of simulation.

### MD analysis

Structural alignments and RMSD measurements were performed using the ptraj program in the AMBER10 program suite. Volume analysis was carried out using POVME [52]. All other measurements were performed using custom scripts and the TCL/TK interface in VMD [53].

### Butyryl-CoA model construction

To determine the placement of CoA, chain A of *Shewanella oneidensis* (PDB:2OAS) was aligned to a single chain of RipA using the MultiSeq tool in VMD [54]. For the crystal structure model, alignment was performed to chain A of the crystal structure. For the MD model, alignment was performed to a conformation sampled from chain A, after 6.96 ns of the simulation elapsed. The butyryl moiety was added to CoA using the Maestro program from Schrödinger (Maestro, version 9.2, Schrödinger, LLC, New York, NY, 2011). Effort was taken to minimize steric conflicts while maintaining the proximity of Glu249, the putative nucleophile, and the butyryl-CoA thioester electrophile. Constraints were then placed on the protein, and butyryl-CoA was minimized during a 1500 step Polak-Ribiere conjugate gradient optimization using GBSA implicit solvent and the 2005 OPLS force field. The minimization was performed in MacroModel v9.8, from Schrödinger (MacroModel, version 9.8, Schrödinger, LLC, New York, NY, 2010).

## Supporting Information

Figure S1 **The reaction mechanism of family I CoA-transferases.**
(TIFF)

Figure S2 **RipA, the N-terminal and C-terminal domains are colored blue and red, respectively.** The proposed active site glutamate, Glu249, Val227 and acetate are in stick representation with carbon, oxygen and nitrogen atoms are colored, yellow, red and blue, respectively. The electron density mesh is colored in blue and acetate and Glu249 fits the density well.
(TIFF)

Figure S3 **Tetramer and monomer RMSDs.**
(TIFF)

Figure S4 **Distance between monomer centers of mass.** A) Distances separating centers of mass of monomers across the dimer-dimer interface. B) Distances separating centers of mass of monomers within each dimer.
(TIFF)

Figure S5 **Fractional contact times and physico-chemical character of the dimer-dimer interface.** (A) The fractional contact times of residues that are within 5 Å any residue that belongs to the monomer across the dimer-dimer interface are mapped to the surface of the protein and coded by color. (B) The physico-chemical character of residues with a fractional contact time greater than 0. Blue is basic, red is acidic, white is nonpolar, and green is polar.
(TIFF)

Figure S6 **Bimodal volume distribution.**
(TIFF)

Figure S7 **Time series of active site volume and Val227 loop extension.** A) Volume time series by monomer. Chains are colored uniquely and labeled. Chain A is red, chain B is blue, chain C is green, and chain D is pink. B) Time series of the distance separating the a-carbon atom of Asn252 from the b-carbon of Val227. Coloring is as in A. The Pearson coefficient between these two series is −0.72.
(TIFF)

Figure S8 **Distribution about the Glu61 CA-CB bond.** The peaks are described in the “dynamics of the putative acyl-binding pocket” section.
(TIFF)

Figure S9 **Proposed tetrameric assembly for** ***E. coli*** **YdiF (PDB code:2AHU).** Each monomer in the dimer is colored in yellow and green, and the crystallographic symmetry axis is marked with a red line and is at the dimer-dimer interface of the tetramer.
(TIFF)

Table S1 **Physico-chemical character at the interface surface and total monomer surface.**
(DOC)

Table S2 **Fractional pair-wise contacts spanning the dimer-dimer interface.** Monomer residues numbers and single letter amino acid codes for monomers from each side of the dimer-dimer interface run along the horizontal and vertical.
(DOC)
