# Supplementary figures and images for: Biochemical, Structural and Molecular Dynamics Analyses of the Potential Virulence Factor RipA from Yersinia pestis
Source: PLoS One. 2011 Sep 26;6(9):e25084. doi: 10.1371/journal.pone.0025084 (PMC3180442; doi:10.1371/journal.pone.0025084)

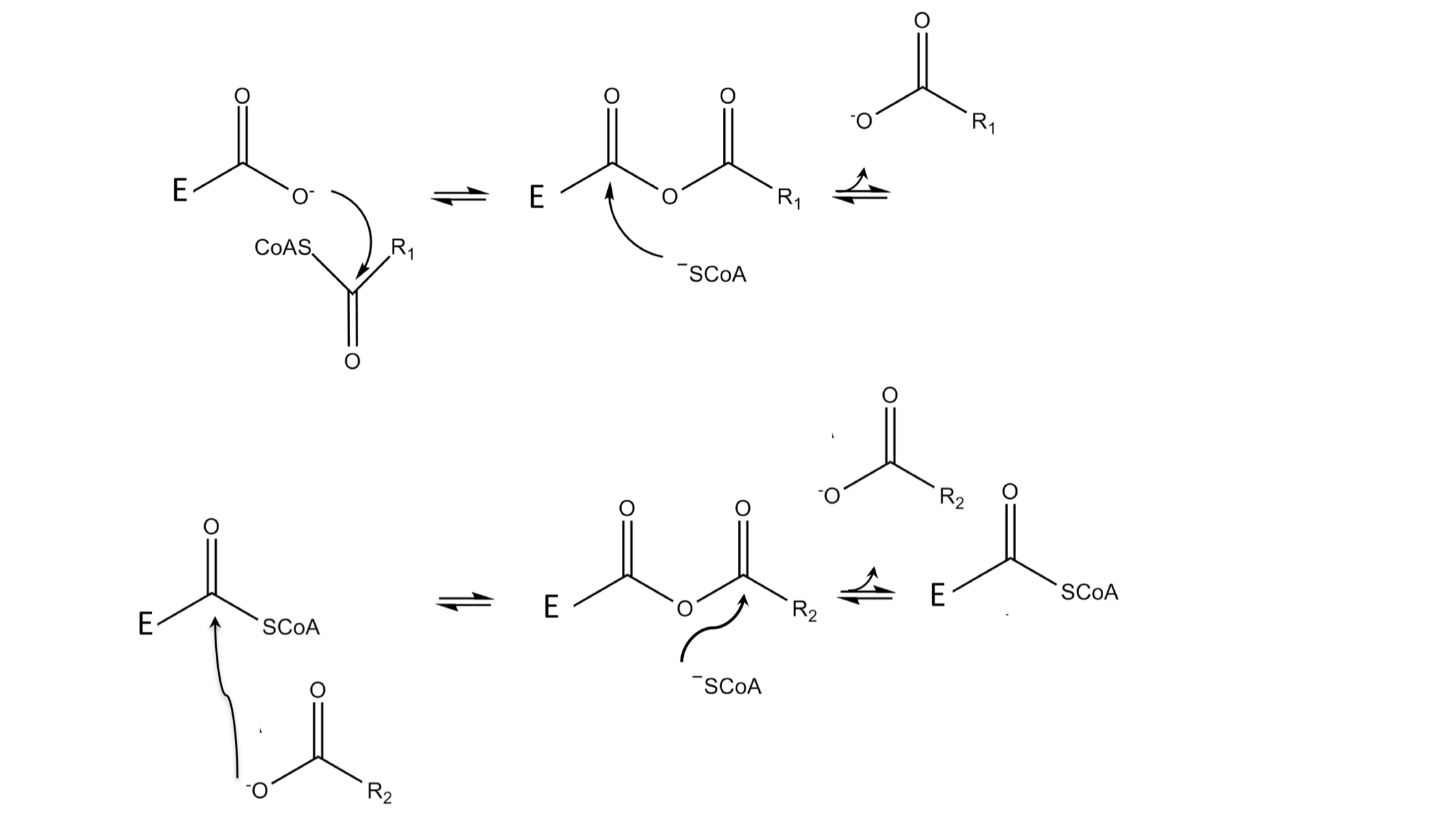

Supplement: Figure S1 — The reaction mechanism of family I CoA-transferases. (TIFF) [file pone.0025084.s001.tif]

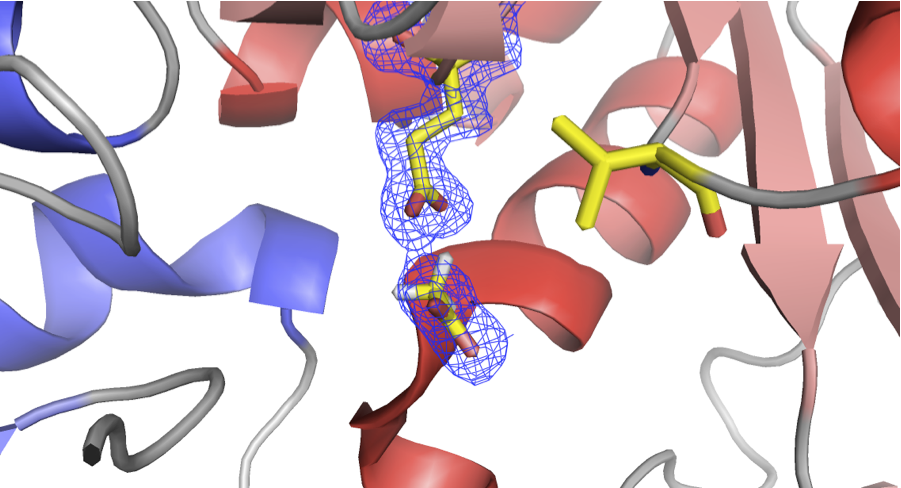

Supplement: Figure S2 — RipA, the N-terminal and C-terminal domains are colored blue and red, respectively. The proposed active site glutamate, Glu249, Val227 and acetate are in stick representation with carbon, oxygen and nitrogen atoms are colored, yellow, red and blue, respectively. The electron density mesh is colored in blue and acetate and Glu249 fits the density well. (TIFF) [file pone.0025084.s002.tif]

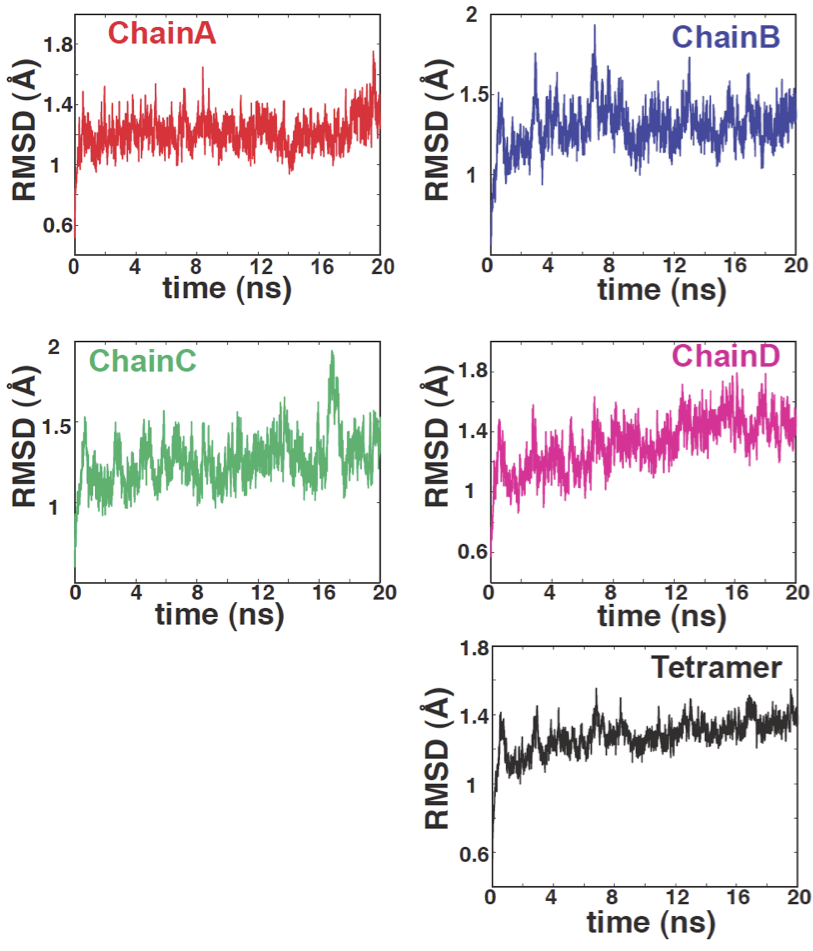

Supplement: Figure S3 — Tetramer and monomer RMSDs. (TIFF) [file pone.0025084.s003.tif]

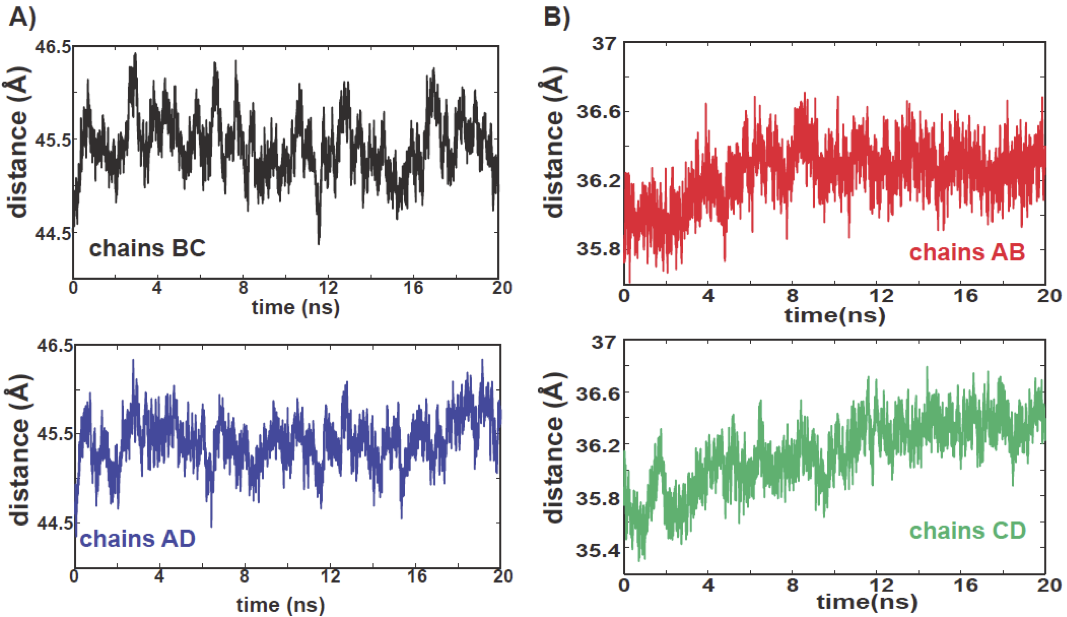

Supplement: Figure S4 — Distance between monomer centers of mass. A) Distances separating centers of mass of monomers across the dimer-dimer interface. B) Distances separating centers of mass of monomers within each dimer. (TIFF) [file pone.0025084.s004.tif]

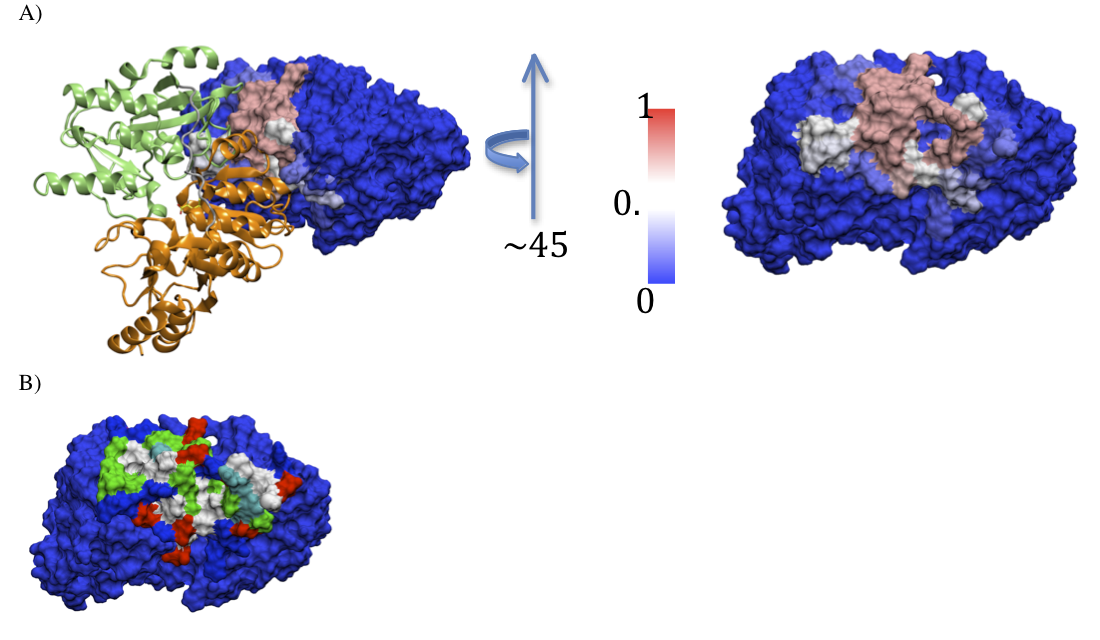

Supplement: Figure S5 — Fractional contact times and physico-chemical character of the dimer-dimer interface. (A) The fractional contact times of residues that are within 5 Å any residue that belongs to the monomer across the dimer-dimer interface are mapped to the surface of the protein and coded by color. (B) The physico-chemical character of residues with a fractional contact time greater than 0. Blue is basic, red is acidic, white is nonpolar, and green is polar. (TIFF) [file pone.0025084.s005.tif]

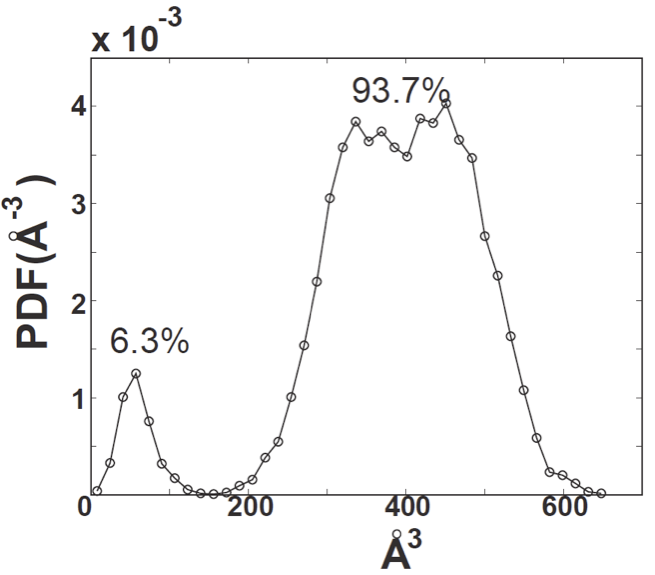

Supplement: Figure S6 — Bimodal volume distribution. (TIFF) [file pone.0025084.s006.tif]

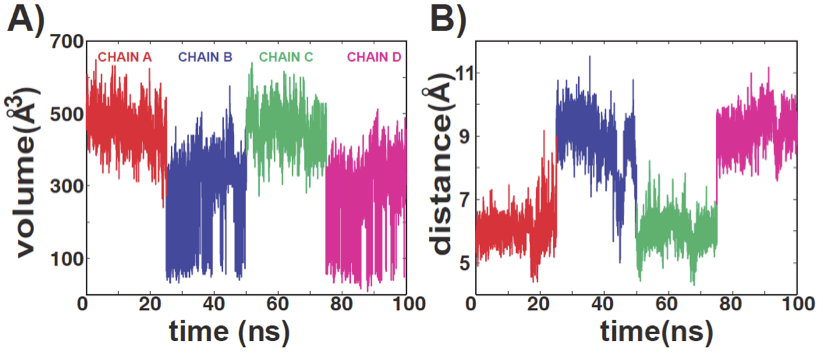

Supplement: Figure S7 — Time series of active site volume and Val227 loop extension. A) Volume time series by monomer. Chains are colored uniquely and labeled. Chain A is red, chain B is blue, chain C is green, and chain D is pink. B) Time series of the distance separating the a-carbon atom of Asn252 from the b-carbon of Val227. Coloring is as in A. The Pearson coefficient between these two series is −0.72. (TIFF) [file pone.0025084.s007.tif]

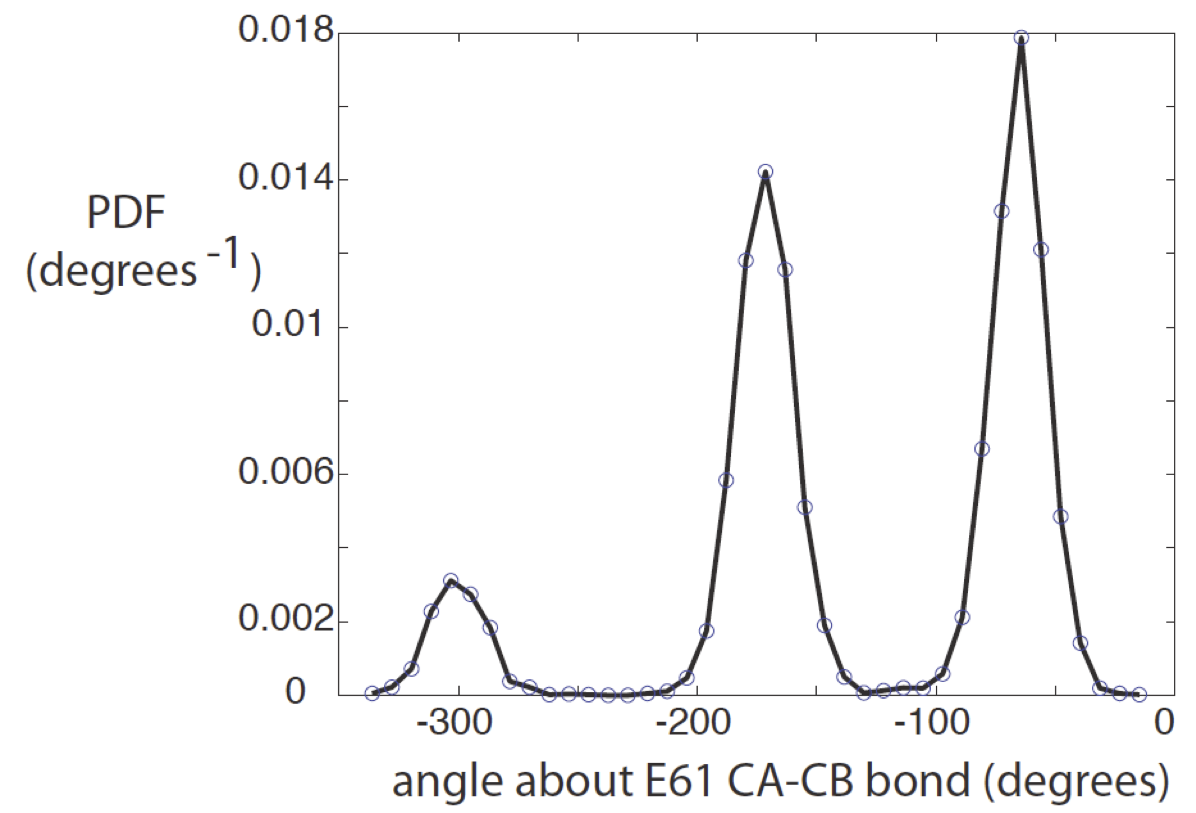

Supplement: Figure S8 — Distribution about the Glu61 CA-CB bond. The peaks are described in the “dynamics of the putative acyl-binding pocket” section. (TIFF) [file pone.0025084.s008.tif]

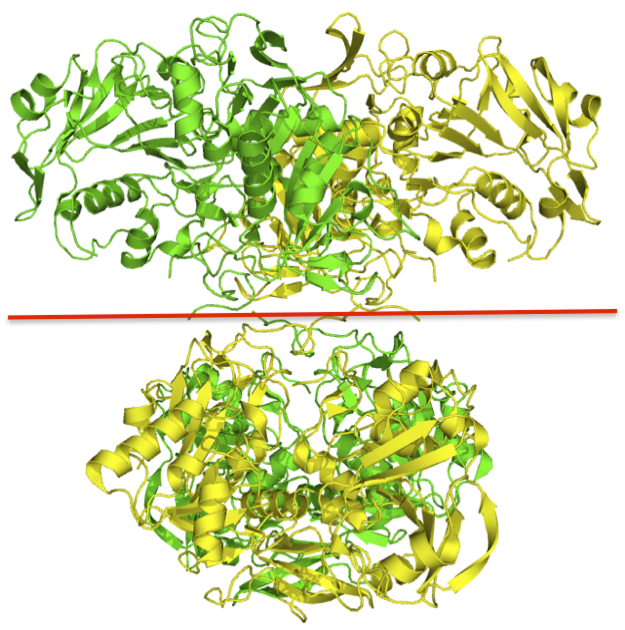

Supplement: Figure S9 — Proposed tetrameric assembly for E. coli YdiF (PDB code:2AHU). Each monomer in the dimer is colored in yellow and green, and the crystallographic symmetry axis is marked with a red line and is at the dimer-dimer interface of the tetramer. (TIFF) [file pone.0025084.s009.tif]
