# Supplementary material for: Biochemical, Structural and Molecular Dynamics Analyses of the Potential Virulence Factor RipA from Yersinia pestis
Source: PLoS One. 2011 Sep 26;6(9):e25084. doi: 10.1371/journal.pone.0025084 (PMC3180442; doi:10.1371/journal.pone.0025084)
Supplement: Table S1 — Physico-chemical character at the interface surface and total monomer surface. (DOC) [file pone.0025084.s010.doc]

| Residue Type | % at the interface surface^*^ | % over the total monomer surface^*^ |
| --- | --- | --- |
| Basic | 38.3 +/-1.0 | 31.7 +/-.54 |
| Acidic | 14.3 +/-0.76 | 18.2 +/-0.37 |
| Non polar | 23.9 +/-1.0 | 30.6 +/-0.54 |
| Polar | 23.4 +/-1.0 | 19.5 +/-0.49 |

*These values are averages, taken over the entire simulation then averaged over each monomer. The standard deviation is also reported
