# Supplementary material for: Biochemical, Structural and Molecular Dynamics Analyses of the Potential Virulence Factor RipA from Yersinia pestis
Source: PLoS One. 2011 Sep 26;6(9):e25084. doi: 10.1371/journal.pone.0025084 (PMC3180442; doi:10.1371/journal.pone.0025084)
Supplement: Table S2 — Fractional pair-wise contacts spanning the dimer-dimer interface. Monomer residues numbers and single letter amino acid codes for monomers from each side of the dimer-dimer interface run along the horizontal and vertical. (DOC) [file pone.0025084.s011.doc]

|  | **D137** | **H139** | **Y141** | **R171** | **Q173** | **A178** | **R275** | **E289** | **Y290** | **N292** | **D293** | **H294** | **P295** | **A296** | **F298** |
| --- | --- | --- | --- | --- | --- | --- | --- | --- | --- | --- | --- | --- | --- | --- | --- |
| **D137** |  |  |  |  |  |  |  |  |  |  | 0.96 |  | 0.80 |  |  |
| **H139** |  |  |  |  |  |  |  | 0.83 | 0.98 | 0.97 | 0.89 | 1.00 | 0.51 |  |  |
| **Y141** |  |  |  |  |  |  |  |  |  |  |  | 0.99 | 0.66 | 1.00 |  |
| **R171** |  |  |  |  |  |  |  |  |  |  | 0.94 |  | 0.61 |  |  |
| **Q173** |  |  |  |  | 0.97 |  |  |  |  |  |  |  |  |  |  |
| **G174** |  |  |  |  |  |  |  |  |  |  |  |  |  |  |  |
| **A178** |  |  |  |  |  |  |  |  |  |  |  |  | 0.66 |  |  |
| **R275** |  |  |  |  |  |  |  |  |  |  |  |  |  |  | 0.85 |
| **E289** |  | 0.78 |  |  |  |  |  |  |  |  |  |  |  |  |  |
| **Y290** |  | 0.96 |  |  |  |  |  |  |  |  |  |  |  |  |  |
| **N292** |  | 0.95 |  |  |  |  |  |  |  |  |  |  |  |  |  |
| **D293** | 0.95 | 0.84 |  | 0.92 |  |  |  |  |  |  | 0.83 |  |  |  |  |
| **H294** |  | 1.00 | 0.99 |  |  |  |  |  |  |  |  |  |  |  |  |
| **P295** | 0.83 | 0.51 | 0.99 | 0.61 |  | 0.98 |  |  |  |  |  |  |  |  |  |
| **A296** |  |  | 1.00 |  |  |  |  |  |  |  |  |  |  |  |  |
| **F298** |  |  |  |  |  |  | 0.58 |  |  |  |  |  |  |  |  |
